# A Game-Theoretical Approach to Multimedia Social Networks Security

**DOI:** 10.1155/2014/791690

**Published:** 2014-04-13

**Authors:** Enqiang Liu, Zengliang Liu, Fei Shao, Zhiyong Zhang

**Affiliations:** ^1^University of Science and Technology Beijing, Beijing 100083, China; ^2^Xidian University, Xi'an 710126, China; ^3^Information Engineering College, Henan University of Science and Technology, Luoyang 471023, China

## Abstract

The contents access and sharing in multimedia social networks (MSNs) mainly rely on access control models and mechanisms. Simple adoptions of security policies in the traditional access control model cannot effectively establish a trust relationship among parties. This paper proposed a novel two-party trust architecture (TPTA) to apply in a generic MSN scenario. According to the architecture, security policies are adopted through game-theoretic analyses and decisions. Based on formalized utilities of security policies and security rules, the choice of security policies in content access is described as a game between the content provider and the content requester. By the game method for the combination of security policies utility and its influences on each party's benefits, the Nash equilibrium is achieved, that is, an optimal and stable combination of security policies, to establish and enhance trust among stakeholders.

## 1. Introduction

Multimedia social networks (MSNs) are currently in the wave of popularity. It allows users to share music, pictures, home movies, blogs, and other digital contents with friends, family, colleagues, and students quickly and easily. In the past few years, MSNs, such as MySpace, Facebook, LinkedIn, Flickr, and YouTube, have become the most convenient online sharing method in sharing of images, videos, audios, and other multimedia contents. Although MSNs make communication between people easier and faster and enhance information dissemination among people, there are also security issues, such as privacy disclosure and copyright disputes. This has, undoubtedly, brought serious harm to the dissemination and development of the Internet information. In response to these security issues, the access control mechanism provides a method that allows selective media contents sharing in MSNs. The access control mechanism determines which users can access what resources and how to use these resources and allows users to selectively share their digital contents. Using access control mechanism on digital rights management, content providers can choose to accept or reject access requests after verifying the access conditions of the digital contents [1, 2].

In the existing MSNs, the access control includes two main types: the relationship-based access control and the trust-based access control.

(*1) Relationship-Based Access Control*. Gates [3] described a new, relationship-based access control security paradigm to meet the needs of Web 2.0. Hart et al. [4] proposed a content- and relationship-based access control system using relationship information in web based social network (WBSN) to represent the authorized agent, which satisfied the key requirements for protecting WBSN resources. However, the system did not achieve the enhanced privacy needs in access control, considered only the direct relationship, and did not take consideration of the node trust in access authorization. In terms of privacy concerns, it focused on privacy protection and data mining techniques and allowed social network analysis for potential sensitive information that had no public disclosure possibility. Park et al. [5] proposed a user-behavior-centric access control framework and identified four core control behaviors: attributes, policies, relationships, and sessions. The proposed online social network (OSN) had the following characteristics. First, in personalized policies, the OSN users had their own security and privacy policies and attributes. Second, the proposed OSN separated the users from resource policies. Third, the proposed OSN supported access control that was independent of the user relationship and sessions that represented actions. It also took into account the enhanced control, which is not referred to in the existing OSN services. Many of the latest literatures on the OSN access control cannot distinguish between sessions and users.

(*2) Trust-Based Access Control*. Ali et al. [6] applied a multilevel security approach, in which trust was the only parameter that was used to determine the security levels of the users and resources. More precisely, each user was assigned a reputation value. The reputation value was a user's average trust level that was specified by other users. However, Ali and his colleagues only considered direct trust relationship without taking into account the indirect trust relationship. Kruk et al. [7], then, proposed a distributed authentication management system based on the second round “friend” relationship to bring out the management of access rights and trust authorization. Wang and Sun [8] proposed a trust-related management framework that included access control policies and a privacy protection mechanism. This mechanism administers the access policies on the data that contain the provable information, enhances the support to the highly complex privacy related policies, and takes consideration of the purpose and obligations. Under this mechanism, the agent can perform access rights on the objects based on relationships, trusts, purposes, and obligations. This mechanism also introduced strategic operations and the concept of policy conflicts and proposed a purpose related access control policy framework. Sachan et al. [1] pointed out that the traditional access control cannot meet the fine-grained access control requirements and the large number of users. To solve this problem, they proposed an efficient bit-vector transform based access control mechanism suitable for MSNs. They converted the content related certificate into an efficient architecture and, then, verified the security, storage, and execution efficiency of the proposed mechanism rough simulations. Villegas [2] proposed a personal data access control (PDAC) scheme. PDAC computes a “trusted distance” measure between users that is composed of the hop distance on the social network and an affine distance derived from experiential data. Zhang and Wang [9] proposed a trust model for social networks. Based on deep analysis of the characteristics of social networks, they developed a computational model for calculating trust in social networks. Carminati et al. [10] proposed a rule-based access control model and used certificate chain as a parameter for calculating trust, so as to realize effective control of content access in social networks.

These studies focused mainly on relationship and trust-based user access control and realized the controllable, safe transmission of digital content in the MSNs. However, in the relationship-based user access control, only the direct relationship is considered, while the indirect relationship between users, the type of relationship, and closeness of the relationships are not considered. In the study of trust-based access control, there was no unified understanding of the trust threshold. The setting of the trust threshold can directly affect the security of digital content and controllable dissemination.

In order to solve these issues in social network access control and to prevent excessive denial to normal access or access to much malicious contents, the approach of adopting security policies through game-theoretic analyses is proposed. Tian and Lin [11] proposed a trust prediction-based game control mechanism for trustworthy networks. This mechanism could not only predict behavior trust level with single trust attribute but also could predict trust level with the multiple trust attributes, so as to help participants to achieve the maximization of utility. Wen et al. [12] proposed game-theoretic model for information dissemination in social networks. This model reflected the influence of human behavior on information dissemination and conceptualized participants' utility function based on different parties' interests. An empirical study indicated that information dissemination can be divided into several stages, and the dissemination speed is limited by the characteristics of each person in the network. Zhang et al. [13] proposed game-based social network access control. For the “nonfriend” type of access users, on the basis of defining user trust and its calculation method, this study conducted game-theoretic analyses by integrating the payoff matrix of both the content provider and the content requester, calculated the hybrid Nash equilibrium, provided decision-making criteria for access control, and finally analyzed the utility of the access control method with examples. None of these three models consider the personalization problem of the content providers' security policy and only conceptualize it as accepting or denying access. In addition, in the participants' utility function, none of the models consider the inherent cost, such as the cost of implementing security policies for the content providers and the cost of malicious access for the access requesters. Zhang et al. [14, 15], for a general digital rights management (DRM) value chain system, proposed a layered analysis of multiparty trust architecture by using game-theoretic analyses of adoption of security policies. Based on formalized utilities of security policies and services, the adoption of security policies with external relativity is described as a game between the content provider, the digital services/providers, and the content requester. Based on the utility of the security policies and their influence on each party's benefits, the Nash equilibrium value was achieved, which is an optimal and stable combination of security policies, thus establishing and strengthening multiparty trust. In order to effectively select and deploy security policies in content sharing scenarios, Zhang et al. [16] introduced the game theory to analyze the influence of security policies that use trusted-computing-enhanced security policy stakeholders. At last, Zhang et al. conducted game-theoretic analyses and swarm simulation. The results indicated that the obtained digital content and security cost had direct impact on the content provider's choice of security policies. In addition, different basic-sharing models, including local, intermediate, and extensive sharing models, will further affect the choice of the content providers. The mixed-sharing model was much more similar to the real content sharing situations. Due to limited power, sharing, and higher security cost, the dynamic security policy is better than the fully enhanced security policies; but with the reduction of more power and enhanced security cost, the latter strategy would be the best and the most stable Nash equilibrium [17, 18].

As noted above, there are a lot of studies about access control issues in MSNs; however, a successful access of multimedia digital content (MMDC) should have the following three factors: security, trust, and benefits. So far, because of the lack of access control in MSNs, the MMDC access is only based on security policies and the related mechanisms. Therefore, how to make a rational use of security policies to maximize the benefits of the participants is worth considering. This paper proposes a game-based security policies adoption approach for MSNs. This system is benefits-centric that enables the participants to find an optimal and stable security policy in MSNs.

## 2. Formalized Game of Security Policies

### 2.1. Two-Party Trust Architecture

Recently, game theory is widely applied in economics, biology evolution, and information technology, especially for the decision-making on information security polices, when multiple stakeholders have their own benefits and strategies moving. The MSN scenario has such characteristics as needed by game theory.

**Figure 1:**
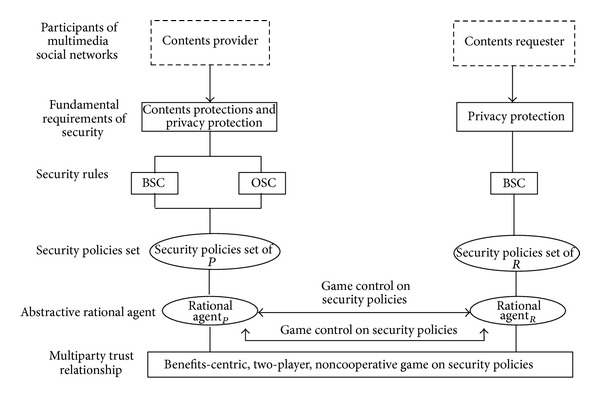
Two-party trust architecture in multimedia social networks.

A general MSN is composed of different stakeholders, such as* P* (content providers) and* R *(content requester). Based on the basic analysis of the trust relationship, two-party trust architecture (TPTA) is the trust architecture between *P* and *R*. This system includes a set of security rules, namely, the basic security rules and the optional security rules. The specific security policies can be achieved by using these security rules. As shown in Figure 1, participants are rational agent (RA), who can, rationally, select and deploy a security policy based on the game theory.

### 2.2. Basic Components

**Definition 1 (party)**

A symbol *℘* denotes personal player participating in content provider and content access; these two roles are interchangeable in MSNs. The *℘* can take different roles in MMDC sharing; the content providers can assume the role of the content requester, while the content requester can also take the content provider role. The formalized *℘* participant is as follows:

(1) ℘={α ∣ stakeholder  accessing  to  contents}MMSN_VauleChainMPTA={P,R,MMDC}.

**Definition 2 (security rules)**

In response to the participants' security requirements, one security rule corresponds to one user attribute constraint, which ensures the security of MMDC. A symbol SR* denotes basic security rules; the other SR denotes optional security rules. Notation of *f*, *w*, *u* denotes an effective factor from factor set *F* influencing benefit of *℘*, the weight value of factor, and a positive/negative utility, respectively. Here, the normalized weight is based on the weight of all of the factors of SR:

(2) security  rule ={SR1∗,SR2∗,…,SRi∗,SR1,SR2,…,SRj}F(srs) ={fsr⁡1,fsr⁡2,…,fsr⁡l} (1≤s≤l),μ(srs) =∑i=1lui(wi∑k=1hwk).

**Property 1 (external relativity of optional security rules)**

If two or multiple optional security rules are from different parties, choose to adopt simultaneously or adopt only one of them according to the needs of participants. The external relativity of these rules is described as follows, in which *ℂ*(*℘*) denotes the base set of *℘*.

(1) If *P* has some strict requirements for MMDC access (*R* must meet all the security rules before accessing the MMDC),
  (3)       Relative_Components={sr1,sr2,…,srp}        ∀i,j(1≤i,j≤p,2≤p≤ℂ(℘))∃s, t(s,t∈{P,R})(sri∈SRs,srj∈SRt,i≠j⟶s≠t).
(2) If *P* has relaxed requirements for MMDC access (*R* only needs to meet any one of the security rules to access MMDC),
  (4)   Relative_Components={sr1,sr2,…,srp}     ∀i,j(1≤i,j≤p,2≤p≤ℂ(℘))∃s,  t(s,t∈{P,R})(sri∈SRs∨srj∈SRt).

**Definition 3 (security rules)**

Sp includes the *P* and *R*'s sp, denoted, respectively, as sp_*P*_ and sp_*R*_. sp_*P*_ is considered as a set of security rules and services; sp_*R*_ includes normal access and malicious access:

(5) $$\begin{array}{c} \text{s}{\text{p}}_{P}=\left\{\text{s}{\text{r}}_{1}^{*}\ldots ,\text{s}{\text{r}}_{i}^{*},\text{s}{\text{r}}_{1},\text{s}{\text{r}}_{2},\ldots ,\text{s}{\text{r}}_{s}\right\}\;\left(0\leq s\leq j\right),\\ \text{S}{\text{P}}_{Pi}=\left\{\text{s}{\text{p}}_{i}^{1},\text{s}{\text{p}}_{i}^{2},\ldots ,\text{s}{\text{p}}_{i}^{\mathbb{C}(\text{S}{\text{P}}_{i})}\right\}\;\left(\mathbb{C}\left(\text{S}{\text{P}}_{i}\right)=2^{j},i\in \left\{P,R\right\}\right),\\ \text{s}{\text{p}}_{R}=\left\{\text{normal},\text{malicious}\right\}. \end{array}$$

**Definition 4 (utility of sp)**

Utility *U*_*P*_ of sp_*P*_ is a sum of utilities of all rules or services involved in sp_*P*_; utility *U*_*R*_ of sp_*R*_ is a sum of utilities of all rules or services involved in sp_*R*_:

(6) $$\begin{array}{c} U\left(\text{s}{\text{p}}_{P}^{}\right)=\overset{i}{\underset{p=0}{\sum }}\mu \left(\text{s}{\text{r}}_{p}^{*}\right)+\overset{j}{\underset{p=0}{\sum }}\mu \left(\text{s}{\text{r}}_{p}^{}\right)+\overset{k}{\underset{p=0}{\sum }}\mu \left(\text{MMDC}\right)\\ U\left(\text{s}{\text{p}}_{R}^{}\right)=\overset{i}{\underset{p=0}{\sum }}\mu \left(\text{s}{\text{r}}_{R}^{}\right)+\overset{j}{\underset{p=0}{\sum }}\mu \left(\text{MMDC}\right). \end{array}$$

### 2.3. Formalized Game of Security Policies

**Definition 5 (rational agent)**

A symbol RA denotes a rational actor aiming at a maximization of benefit and makes a decision on adopting a certain security policy. In TPTA, there are two RAs with respect to two parties, namely, RA_*P*_ and RA_*R*_.

**Definition 6 (payoff of RA)**

In TPTA, a payoff RA denotes the acquired benefits from security policies set. It is the carry for RA adoption of security policies. Benefits include two aspects: RA or changes of RA.

**Definition 7 (two-party game)**

Two-party game *G* of security polices denotes a process of making decision on effective and rational adoption of security policies that have effect on benefit of the opposing parties. To achieve utility maximization and balance, the game is depicted by a set of three tuples as 〈*℘*, sp, payoff〉. SP represents the security policies set:

(7) $$\begin{array}{c} G=\left\{\langle \text{R}{\text{A}}_{i},\text{S}{\text{P}}_{i},\text{Payoff}\left(\text{R}{\text{A}}_{i},\text{R}{\text{A}}_{-i}\right)\rangle \;|\;i=\left\{P,R\right\}\right\}. \end{array}$$

**Definition 8 (Nash equilibrium under policies combination)**

For any RA, when adopting a security policy, sp*acquires greater benefit than the benefit acquired by choosing any other sp; the combination of each RA's sp* is considered as a balance of payoffs by adopting relatively dominant security policies:

(8)  Payoff(RAisp∗,RA−isp∗)≥Payoff(RAispj,RA−isp∗)j∈SPi,j≠∗, i∈{P,R} (−i∈{P,R},−i≠i),

where (sp_P_*, sp_R_*) is a relatively dominant pure policies combination.

### 2.4. Game of Security Policies in Two Scenarios

**Theorem 9 (two parties both change game in content access)**

Content access is a general scenario in MSNs. In this scenario, the adoption of security policies is considered to be a particular game process in which both *P* and *R* change simultaneously.

**Proof**

In TPTA, according to RA_*P*_ and RA_*R*_ in Definition 5, denote their security policies combinations as SP_*P*_ and SP_*R*,_ respectively. Game was further formalized as *G*_acquisition_ =  {〈RA_*i*_, SP_*i*_, Payoff(RA_*i*_, RA_−*i*_)〉}, in which *i* = {*P*, *R*}. For MMDC access,* P* needs to set up security rules for* R*'s MMDC access, that is, choosing a particular sp from SP. Under normal circumstances, the process of content access has timing characteristics; after RA_*R*_ requests MMDC access to RA_*P*_, RA_*R*_ should meet the access control policies. However, when each RA adopts and initializes SP, they do not know other RA's changes of sps. In addition, during the content transaction, the setting of the MMDC security polices in MSNs cannot be changed. Therefore, the change process of RA in security policies is a simultaneous change of the game, rather than a continuous change of the game.

**Theorem 10**

The trust values of the content providers' benefits and those of the content requesters' benefits are proportional.

**Proof**

Based on the utilities of the content provider and the content requester in Definition 4, the trust values of content requesters *R*_*i*_ and *R*_*j*_ are* i*,* j* assuming that* i* <* j*. Because the larger the trust value, the larger the* i*,* j *values; therefore, the trust value of *i* is larger than that of *j*. The larger the user trust value, the larger the *μ*(MMDC) value, the greater the utility and therefore the larger the *P* and *R* benefits.

*Deduction 1 (repeated game in content access scenario)*. When several content access sessions are carried out, the participants in MSNs will choose to reactivate a game in order to select a security policy. The new game can be seen as a repetitive game, which is based on the process and results of the previous game, and get a new equilibrium.

**Proof**

In a given scenario, as the access to content increases, the adoption of security policies will change accordingly. When RA_*P*_ and RA_*R*_ select security policies again, a repeated game will happen, combined with sessions of the previous game and transaction to obtain a new security policies combination, which is called a new Nash equilibrium.

## 3. Game-Theoretic Analysis of Typical Security Strategy

In an access control model of multimedia social network which has universal significance, each party has a security strategy set and practical choice set representing moving in content access. Some typical *P* and *R* security strategies are listed in Section 2.1. The following two sections cite the security benefits of all security rules, effective strategy combinations, and participant benefits, respectively. A strategy selection example is finally analyzed.

### 3.1. Typical Security Strategy

In this study, some typical security strategies are presented. A real access control for multimedia social network may include but is not limited to these strategies. In Definition 2 in Section 2.1, some security rules that can meet the security demand of any party are mentioned first, and then security strategy set can be easily derived.

The security rules of two participants include relationship type (RelT), depth (Dep), compactness (*C*), and trust (*T*).

Similarly, since the SR set of *P* can be denoted as {G*, Dep*, C*, T*}, the security strategy set is {general security strategy, enhanced security strategy}. The enhanced security strategy is *k*_1_ G* + *k*_2_Dep* + *k*_3_ C* + *k*_4_ T*, where *k*_*i*_  (*i* = 1,2, 3,4)∈{0,1},  ∑_*i*=1_^4^ *k*_*i*_ ≠ 0, denoted by sp_*p*_.

For the access into MMDC, there are two types of* R*, normal access (NA) and malicious access (MA). Therefore, the security strategy set is {MA*, NA*}.

By the typical security strategies and related SR analysis above, the utility impact factor, weight, and the utility of SR will be introduced in this section. As SR* cannot change the utility of sp, only the utility of SR is considered here.

### 3.2. Effective Strategy Combination and Its Utility

**Figure 2:**
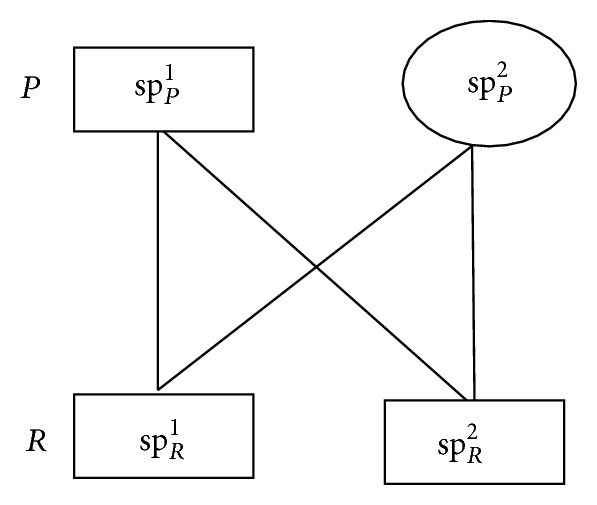
Security strategy combinations.

Since there are two security strategies for each party, there are 4 possible strategy combinations in the game.  Figure 2 describes the security strategy combinations of participants, where sp_*i*_  (*i* ∈ SP, SP) denotes the strategy mentioned in the subgraph.

The benefits of content provider and content requester are defined as follows.

The symbol *U*_*P*_^Benifit_NA^ denotes the fact that when the content provider implements the general safety strategy and the content requester adopts the normal access, the content provider may obtain normal average benefit, such as the rise of the number of friends, increase of attention degree, and acquisition of the information of content requester.

The symbol *U*_*P*_^Damage_MASuccess^ > 0 denotes the possible average amount of loss after the content provider implements general safety strategy and content requester adopts the malicious access, such as the multimedia digital content of the content provider being forwarded casually. Another maliciousness includes impersonating user identity using the content provider's information.

The symbol *U*_*P*_^Damage_NA^ > 0 denotes the possible average amount of loss of content provider when the content provider implements the enforced security strategy and the content requester accesses normally, such as declining normal user accessing so that the social network resource is not fully used and the loss of no cooperation caused by distrust between the two parties.

The symbol *U*_*P*_^Cost^ > 0 denotes the cost of deploying security strategy for content provider, such as the increase of time expenditure. Consider *U*_*P*_^Cost^ = *C*_0_(*k*_1_ G* + *k*_2_Dep* + *k*_3_ C* + *k*_4_ T*).

The symbol *U*_*R*_^Benifit_NA^ denotes the average benefit obtained by the content requester when the content requester accesses normally and content provider implements the enforced security strategy, such as rise of the number of friends, increase of attention degree, and promotion of digital content.

The symbol *U*_*R*_^Benifit_MASuccess^ denotes the excess benefit obtained by content requester when the content requester adopts malicious behaviors and content provider implements general security strategy, such as distributing the multimedia digital content casually and disclosing private information without permission.

The symbol *U*_*R*_^Cost^ > 0 denotes the cost of attacking the security strategy platform by the content requester.

The symbol *U*_*R*_^Punish^ > 0 denotes the punishment that may be given to the content requester adopting malicious behaviors, such as decreasing the trustworthiness of content requester, suspending the access right to social network for the content requester, or suing the content requester, where *U*_*R*_^Benifit_MASuccess^ > *U*_*P*_^Cost^.

First, we analyze the gain and loss of the benefits of both content provider and requester. If the content requester accesses normally and the content provider implements the enforced security strategy, then the content requester and provider will both benefit and their benefits are denoted as *U*_*P*_^Benifit_NA^ and *U*_*R*_^Benifit_NA^, respectively. If the content requester accesses maliciously and the content provider implements the general security strategy, then the loss of content provider is *U*_*R*_^Benifit_NA^, while the benefit of content requester contains an excess benefit *U*_*P*_^Cost^ obtained by malicious access, in addition to the normal average benefit *U*_*R*_^Benifit_NA^. However, the content requester may be subjected to a punishment *U*_*P*_^Cost^ if adopting malicious access. If the content provider implements the enforced security strategy, then there will be neither benefit nor loss but only the cost of implementing the enforced security strategy *U*_*P*_^Cost^. Based on Definition 4 and Figure 2, the payoff matrix of participants under multiple combinations is as follows:

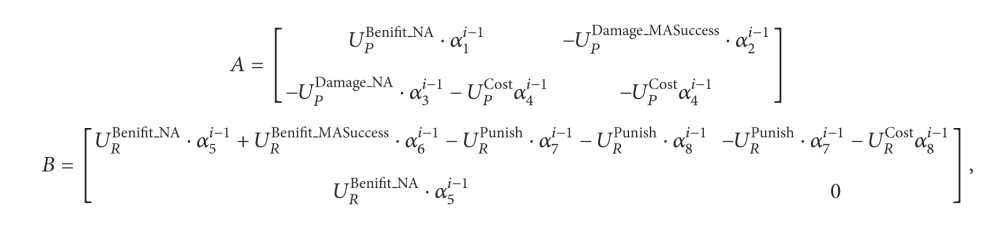
(9)
where *α*_*i*_  (*i* = 1,2,…, 8) > 1 is the parameter factor, mainly used to adjust the ratio of user benefit to punishment. The setting of this value is based on requirements of the decision makers. The benefit matrices* A* and* B* denote that the user's benefit or loss is closely related to his/her attributes and is proportional to the trustworthiness. The reason why the content requester chooses malicious access is that it is believed that the benefit obtained by malicious access is larger than that by normal access; that is, the user is rational. However, the content provider in social networks increases his or her attention degree and maximizes the benefit by making more friends, which means that the content provider is also rational. Supposing that the probability of content provider implementing the general security strategy is *x*, then the probability of implementing enforced security strategy is 1 − *x* and the mixed strategy for the content provider is *P* = (*x*, 1 − *x*). Similarly, supposing that the malicious access probability of content requester is *y*, then the probability of normal access is 1 − *y* and the mixed strategy of content requester is *R* = (*y*, 1 − *y*). Based on Definition 4 and Figure 1, the benefit obtained by the participants under multiple combinations is as follows:

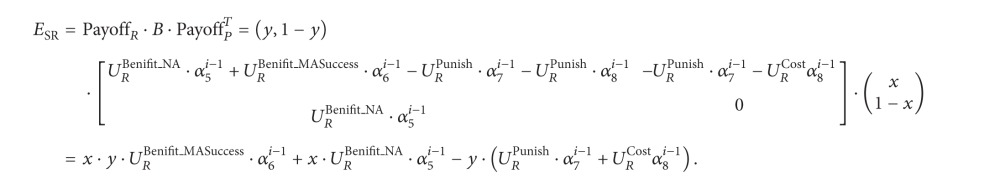
(10)By taking the partial derivative of the above equation with respect to *y*, the condition for the content provider getting the optimal strategy is

(11) ∂ER∂y=x·URBenifit_MASuccess·α6i−1−(URPunish·α7i−1+URCostα8i−1)=0.

Hence, there is

(12) $$\begin{array}{c} x^{*}=\frac{U_{R}^{\text{Punish}}\cdot \alpha_{7}^{i-1}+U_{R}^{\text{Cost}}\alpha_{8}^{i-1}}{U_{R}^{\text{Benifit}\_\text{MASuccess}}\cdot \alpha_{6}^{i-1}}; \end{array}$$

that is to say, P* = (x*, 1 − x*) is the optimal strategy for the content provider.

It can be seen from the result of observation and analysis that the accepting probability of content provider is only related to the benefit and payment of the user. By increasing the punishment for malicious access from the content requester, increasing the cost of attacking security strategy by requester, and decreasing the benefit obtained by successful malicious accesses from the content requester, the probability of content provider adopting the general security strategy can be improved and the normal operation of the social network can be promoted. When the content provider adopts general security strategy in the probability of *x* > x*, the content requester can obtain benefit by normal access; otherwise, the optimal strategy for the content requester is to adopt the normal access strategy. The strategy can only be used to determine at what probability the content provider should accept the access and to select the parameters for decision makers in a macroscopic way. It still depends on the attributes of the content requester and relevant history when it comes to a specific access. A rational content requester seeks a method to maximize his/her own payment to play the game. Therefore, the one that can meet the demand and enable both parties to keep a stable state is the mixed strategy Nash equilibrium, which is the lowest condition acceptable for the content provider. The benefit function of the content provider is expressed as

(13) EP=PayoffP·A·PayoffRT=(x,1−x)·[UPBenifit_NA·α1i−1−UPDamage_MASuccess·α2i−1−UPDamage_NA·α3i−1−UPCostα4i−1−UPCostα4i−1]·(y1−y)=x·y(UPBenifit_NA·α1i−1+UPDamage_MASuccess·α2i−1  +UPDamage_NA·α3i−1)  −x(UPDamage_MASuccess·α2i−1−UPCostα4i−1)  −y UPDamage_NA·α3i−1−UPCostα4i−1.

By taking the partial derivative of the above equation with respect to *x*, the condition for the content requester getting the optimal strategy is

(14) ∂EP∂x=y(UPBenifit_NA·α1i−1+UPDamage_MASuccess·α2i−1+UPDamage_NA·α3i−1)−(UPDamage_MASuccess·α2i−1−UPCostα4i−1)=0.

Hence

(15) y∗=(UPDamage_MASuccess·α2i−1−UPCostα4i−1)×(UPBenifit_NA·α1i−1+UPDamage_MASuccess·α2i−1+UPDamage_NA·α3i−1)−1,

where R* = (y*, 1 − y*) is the optimal strategy for the content requester.

It can be seen from the observation result that the mixed strategy Nash equilibrium for content requester gives an uncertain game-theoretic result to the user. Illegal user is not able to get the payoff matrix and decision probability and therefore is unable to judge how the content provider will process the request. These users can obtain the payoff matrix and decision probability by illegal means, but how the content provider will make decision is not certain.

### 3.3. Dynamic Strategy Control Based on Mixed Strategy Nash Equilibrium

In the above section, the mixed strategy Nash equilibrium for the content provider and requester is calculated, and the issue of user controlling strategy probability is presented. However, it is not certain what the decision will be each time. Besides, it is necessary to decide by combining with the strategy selected by the content requester. This is due to the fact that the attributes and decision probabilities of different content requesters are different and the game controlling strategy depends on the game-theoretic analysis of the two parties, instead of the strategy inference of one party. Hence, the content provider needs to adjust the strategies according to the decision probability of himself/herself and that of the content requester and the requirement of his/her decision probability.

P* = (x*, 1 − x*), R* = (y*, 1 − y*), while the requirement by the content provider on the strategy probability of the content requester is *R*_0_ = (*y*_0_, 1 − *y*_0_).

1. The strategy requirement by the content provider is strict; that is, *y*_0_ ≥ y*:* P* adopts enforced security strategy to increase x*.
2. The strategy requirement by the content provider is strict; that is, *y*_0_ < y*:* P* does not need to increase x* and the general security strategy can be adopted.

## 4. Use Cases Analyses

### 4.1. Background

In multimedia social network, the content provider distributes the multimedia digital content and content requester can ask to access the multimedia digital content. When all attributes of the content requester satisfy the requirement of the access control model of multimedia social network platform, the requester can access the digital content. However, after some content providers access the digital content, they casually distribute the multimedia digital content and disclose the private information without permission to seek illegal benefits. In order to prevent such malicious access behaviour, the multimedia social network platform will adopt certain punishment methods, such as declining users to access digital contents. However, mistakenly refusing normal users to access multimedia digital content is not beneficial to the promotion of digital content and drawing attention, while no access control will not achieve the purpose of preventing malicious access, which will damage the interests of the content provider. By using the proposed mixed strategy, the content provider can avoid malicious access from the users and accept normal access.

### 4.2. Use Cases Game Decision on Security Policies

The parameter factors of game-theoretic analysis *α*_*i*_  (*i* = 1,2,…, 8) are 1, 1.1, 1, 1.1, 1.2, 1.1, 1.2, and 1.1, respectively. The assumed values of other parameters are shown as the second to eighth columns in Tables 1 and 2. By substituting the above parameters into (6) and (3), the probability of content requester adopting malicious access, y*, and that of content provider adopting the general security strategy, x*, can be calculated. The y* and x* can be calculated according to the data in the example. The content provider can make decision based on the dynamic strategy control rule of mixed strategy Nash equilibrium. For multimedia social network application platform, two typical scenes are set up.

*Scene 1*. The level of the relationship between content requester and content provider is 1, set to be 1. *C*_0_ is set to be 1, *k*_*i*_ as 1, depth as 1, closeness as 68, and trustworthiness as 1.

*Scene 2*. The level of the relationship between content requester and content provider is 1. *C*_0_ is set to be 1, *k*_*i*_ as 1, depth as 1, closeness as 98, and trustworthiness as 1.

Based on the above mentioned scenes and use cases, the value requirement of *R* from *P* is given as (0.66, 0.34). According to the value of each parameter in Table 1, it can be calculated that* P* (general security strategy, enforced security strategy) of Scene 1 = (0.75, 0.25),* R* (malicious access, normal access) = (0.68, 0.32). At this time,* P* needs to adopt the enforced security strategy. In Scene 2,* P* (general security strategy, enforced security strategy) = (0.68, 0.32),* R* (malicious access, normal access) = (0.65, 0.32). At this time,* P* only needs to adopt the general security strategy.

The benefit and punishment obtained by the content requester increase with the increase of trustworthiness and closeness and decrease with the deepening of the relationship. With the increase of trustworthiness and closeness of content provider and the decrease of relationship depth, the probability of content requester adopting malicious access is decreasing, while the probability of content provider adopting the general security strategy is increasing. This is in accordance with the actual practice on the social network. The content provider can implement the access control based on the mixed strategy Nash equilibrium between the two parties to further adjust the probability of adopting the corresponding strategy. However, the content requester does not know which strategy the content provider will adopt, and the cost of adopting malicious access and the received punishment is far greater than the benefit obtained from successful malicious access. Hence, the provider requester will not adopt the malicious access strategy easily.

### 4.3. Discussions

The decision-making model and method for adopting of security policies are firmly based on the game theory and its applications on information security, so it is complete and robust. Besides, it has also flexibility due to an ability to represent the game on multiparticipant and multisecurity policies, not only two parties and two strategies. The proposed approach to decision has significant advantages, including effectiveness on MSNs security policies combination realization and deployment, convenience on the least overhead of security management, and benefits and productivity for contents providers owing to wider contents access and sharing in MSNs.

## 5. Conclusions 

This paper proposed a game-based analysis on security policies to obtain an optimal combination of security policies for content access in MSNs, thus achieving utility maximization between users. For this reason, this study created the TPTA between the content provider and the content requester. And then, we proposed a typical game-theoretic control of security strategy, obtained the mixed strategy Nash equilibrium based on security attribute of the user, and analyzed a practical example. In this study, the strategy selection under the existing access control mechanism of social network is addressed. A game-theoretic analysis method is provided for the selection of security strategy by the content provider and for the protection of multimedia digital content. In the future, the research challenge will focus on an in-depth consideration to effectively and rationally deploy security policies by the MSNs game-theoretic analysis of security strategy under content sharing conditions, so as to improve the security, credibility, and flexibility of the real MSNs applications and services. In general, the novel game-theoretical model for MSNs is also suitable for the same scenarios and services where multiple stakeholders have their own benefits and strategies choices, including general social media network and applications.

## Figures and Tables

**Table 1:** Parameters settings of the example on Scene 1.

	Parameters			
	*U*_*P*_^Benifit_NA^	*U*_*P*_^Damage_NA^	*U*_*P*_^Damage_MASuccess^	*U*_*P*_^Cost^
1	100	100	600	70
2	150	150	800	100

**Table 2:** Parameters settings of the example on Scene 2.

	Parameters			
	*U*_*R*_^Benifit_N*A*^	*U*_*R*_^Benifit_MASuccess^	*U*_*R*_^Cost^	*U*_*R*_^Punish^
1	100	500	50	300
2	180	700	50	350
